# Association between altered baroreflex control and pain intensity in fibromyalgia

**DOI:** 10.1186/s12883-025-04566-x

**Published:** 2025-12-05

**Authors:** Christoph Best, Ana L. Sayegh, Anna Mueck, Julia Emde, Kati Thieme, Heidrun H. Krämer

**Affiliations:** 1 Department of Neurology, Philipps-University, Marburg, Germany; 2 Department of Neurology, Justus Liebig University, Klinikstraße 33, Giessen, 35392 Germany; 3 Department of Medical Psychology, Philipps-University, Marburg, Germany

**Keywords:** Fibromyalgia, Microneurography, Lower body negative pressure (LBNP), Muscle sympathetic nerve activity (MSNA), Pain control, Autonomic nervous system

## Abstract

**Background:**

The role of the sympathetic nervous system dysfunction in fibromyalgia (FM) is unclear. A large series of studies has shown that various abnormalities of the autonomic nervous system are present in fibromyalgia and contribute at least in part to the patients’ complaints and symptoms.

**Methods:**

In FM patients (*n* = 10) and healthy controls (HC; *n* = 11) pain intensity (numeric rating scale; NRS), cardiac baroreflex sensitivity (BRS) and muscle sympathetic nerve activity (MSNA) via microneurography at rest and during baroreflex stimulation (lower body negative pressure; LBNP) were assessed.

**Results:**

Resting sympathetic activity was higher in FM at baseline (FM patients: 31.1 ± 2.3 bursts/min; HC: 24.3 ± 1.5 bursts/ min, F = 5.736; *p* = 0.028) and during baroreflex stimulation (F = 5.057; *p* = 0.044). Pain intensity correlated with MSNA activity (*r* = 0.760; *p* = 0.018) and showed a tendency to a negative correlation with BRS (*r*=-0.641; *p* = 0.063).

**Conclusion:**

Changes in the baroreflex circuit with increased sympathetic outflow are associated with increased pain perception in FM.

**Supplementary Information:**

The online version contains supplementary material available at 10.1186/s12883-025-04566-x.

## Background

Fibromyalgia (FM) is a multi-symptomatic disorder characterized by generalized pain, fatigue and unrefreshed sleep. Despite advances, the pathophysiology of FM is still under investigation. Possible underlying factors include changes in pain processing, altered stress response, neurotransmitter levels in the hippocampus and elsewhere, small nerve fiber reduction and autonomic nervous system dysfunction [1–4].

The baroreflex circuit, among other mechanisms, regulates the blood pressure via changes of heart rate and resistance of blood vessels. In humans, brainstem regions are associated with this process [5–7]. At rest, a complex network is linked to muscle sympathetic nerve activity (MSNA) [5] reflecting the activity of vasoconstrictors, which stabilize blood pressure. The dorsal medial nucleus tractus solitaries (NTS) represents the first relays station in baroreflex control and has also impact on the regulation of pain, sleep and catecholamins [8, 9]. It has been show that increased baroreflex sensitivity (BRS) in healthy subjects correlates with lower pain sensitivity, resulting in higher pain thresholds [10]. In patients with chronic pain, e.g. chronic back pain, the correlation between BRS and pain perception inversed [11, 12]. BRS changes are associated with chronic pain development and maintenance in different conditions [13], including rheumatoid arthritis, fibromyalgia and other chronic pain disorders [14]. Moreover, in rheumatoid arthritis, it is agreed that increased pain intensity is linked to elevated MSNA [13]. In FM patients, an inversed correlation between BRS and pain perception was detected. Reduced BRS reactivity was correlated with increased pain perception [15]. Nevertheless, it remains unclear whether changes in BRS induce altered pain perception, or whether altered BRS activity is a consequence of chronic pain.

Possible CNS sites of interaction of pain and autonomic nervous system are the dorsal anterior cingulate cortex (dACC) and periaqueductal gray (PAG), the right dorsolateral prefrontal cortex (dlPFC), left anterior insula (AI), and the precuneus. A bi-directional interaction between the autonomous and pain processing system within these cortical regions is proposed [16].

Analyzing the interaction of pain and MSNA in FM patients, inconsistent results have been reported: Elam and coworkers demonstrated that MSNA levels were not elevated in FM patients at rest compared to healthy controls (HC). Furthermore, after various sympathetic stimulation maneuvers, there was no significant difference in subsequently increased sympathetic activity, as measured by MSNA, between FM patients and healthy controls [17]. Furlan and colleagues were able to demonstrate significantly higher MSNA levels in FM patients at rest compared to healthy controls. After sympathetic stimulation using a tilt test, no increased MSNA levels were observed in FM patients in contrast to healthy controls. The lack of an increase in MSNA, i.e., a failure to increase the sympathetic innervation of vasoconstrictors, then resulted in an increased rate of syncope [18]. Rizzi et al. also demonstrated elevated MSNA levels in FM patients under resting conditions compared to healthy controls. After tilt test stimulation, however, no significant increase in MSNA level was observed. Rizzi and coworkers conclude that a bidirectional relationship between pain intensity and sympathetic activation as measured by MSNA is possible. Intensified pain could trigger elevated MSNA levels, which could then centrally induce increased BRS and thereby reduce pain thresholds [19].

In this study, we examined BRS (as measure of the autonomic system’s reflex capacity in response to changes in blood pressure) as well as sympathetic nerve activity by MSNA (characterizing direct tonic sympathetic outflow at rest) in FM using microneurography. We explored how sympathetic activity differs at baseline and during lower body negative pressure (LBNP) stimulation. Autonomic dysfunction has been described in fibromyalgia (FM), but its relationship with pain perception was reported inconsistent. We therefore aimed to investigate the association between autonomic activity, which was evaluated by MSNA and BRS, and pain perception to better understand the role of sympathetic nervous system activity in the pain persistency of FM pain.

## Materials & methods

### Participants

The prospective, monocentric pilot study included only female FM patients aged 18 to 65 years. Ten female fibromyalgia patients were recruited from the outpatient clinic of the Department of Medical Psychology, Phillips-University, Marburg. The diagnosis of fibromyalgia syndrome was established in accordance with the classification criteria of the American College of Rheumatology [20–22].

Sex and age-matched volunteers (healthy controls) were recruited from the staff and relatives of the Department of Neurology, Justus-Liebig-University, Giessen. None of the participants had a history of autonomic disease, chronic pain or took medication interfering with the autonomic nervous system, or analgesics. All participants negated drug or alcohol abuse. The HC group was pain free at study entrance. All patients prior to study inclusion underwent nerve conduction studies of the peroneal nerve, ruling out neuronal damage before inserting microneurography electrodes. For demographic data of the participants see Table 1.

**Table 1:** Physical, clinical and autonomic characteristics at baseline in patients with FM and healthy controls. Displays biographical, clinical and baseline data of FM patients and healthy controls

Variables	FM (*n* = 10)	Controls (*n* = 11)	Statistics*
Age (years)	57 (IQR: 53.5–63)	56 (IQR: 50–63)	F = 0.046; *p* = 0.832
Sex	10 women	11 women	n.a.
Pain intensity (NRS)	4.89 ± 0.66	0	n.a.
HR (beats/min)	73.3 ± 2.1	70.9 ± 4.0	F = 0.321; *p* = 0.730
Systolic BP (mmHg)	139.7 ± 7.2	137.7 ± 2.2	F = 0.059; *p* = 0.811
Diastolic BP (mmHg)	79.0 ± 2.5	84.7 ± 0.8	F = 3.449; *p* = 0.084
Baseline MSNA frequency (bursts/min)	31.1 ± 2.3	24.3 ± 1.5	F = 5.736; *p* = 0.028
BRS (ms/mmHg)	5.0 ± 0.5	21.1 ± 1.8	F = 14.791; *p* < 0.001

*FM* fibromyalgia, *HR* heart rate, *BP* blood pressure, *NRS* numeric rating scale

*one-way ANOVA within the groups FM and healthy controls

### Exclusion criteria

Exclusion criteria for the participants (FM and HC) were cardiac arrhythmias, essential hypertension, any neurological abnormalities or known neurological diseases. All participants underwent a detailed clinical neurological examination before inclusion. Furthermore, serious inflammatory rheumatic, nephrological, oncological or psychiatric diseases accounted for a study exclusion. Finally, to exclude interferences between medications and our results, the participants were excluded or at least paused medications that interfered with the sympathetic nervous system, e.g. beta blockers, opioids or statins. The washout phase was set to 72 h before the experiment. Also known allergies to band aid and left-handedness lead to non-inclusion. A total of 15 FM patients were screened for inclusion, one refused to participate in the microneurographic examination and was therefore excluded, and four patients presented with active comorbid psychiatric disorders and were therefore no enrolled into the study.

### Pain rating

Mean pain intensity over the last week was assessed in all patients using a numeric rating scale before the experiment started (NRS, 0–10). The Numerical Rating Scale (NRS) for pain is a self-report scale ranging from 0 to 10. 0 represents ‘no pain’ and 10 represents ‘the worst pain imaginable’. Patients select the number that best reflects their current level of pain. The NRS is a simple, validated and sensitive instrument for measuring pain intensity [23, 24].

All patients and healthy controls underwent MSNA recording via microneurography during rest and stimulation via lower body negative pressure (LBNP).

### Experimental measurements

Resting cardiovascular parameters (heart rate (HR), systolic (SBP) and diastolic blood pressure (DBP) as well as BRS) were evaluated after resting 15 min.

The participants rested in the bottom shell of a custom-made LBNP chamber. HR was monitored continuously on a beat-to-beat basis through lead II of the electrocardiogram. A microelectrode (0.2 mm wire with a 5 μm tip) was placed into the peroneal nerve to investigate postganglionic multiunit MSNA [25]. The signals were amplified (x 100000) and band-pass filtered (700–2000 Hz). Sympathetic activity was rectified and integrated (time constant 0.1s) to obtain a mean voltage display.

After the MSNA recording site was established, a baseline period of 10 min was recorded. Thereafter, the top shell of the LBNP chamber was hermetically sealed. LBNP stimulation was performed for 10 min [7]. Suction was performed with − 40 mmHg, quantified and monitored by a pressure gauge.

### Baroreflexsensitivity (BRS)

Baroreflex sensitivity was calculated as a ratio of blood pressure and heart rate. The spectral method was applied (http://www.nevrokard.eu/maini/brs.html). Two frequency band were analyzed, a low-frequency band between 0.04 Hz and 0.15 Hz and a high-frequency band between 0.15 Hz and 0.5 Hz. We calculated the square root of the frequency power values between HR and systolic arterial pressure. Using the high-frequency BRS band, this then expressed the cardiac BRS [14]. Continuous BP were evaluated with a photoplethysmographic device (Finometer™ PRO) attached to the middle finger of the left hand. HR was continuously measured through ECG lead II.

### Data analysis

MSNA signals were analyzed offline [7]. The setup allowed the automatic identification of MSNA bursts, followed by visual confirmation (AS, HHK). Muscle sympathetic bursts were expressed as burst frequency (BF, bursts per minute). BF is a useful measure of the degree of direct sympathetic activity reaching the effector organs [26]. In addition, BF was normalized to baseline to evaluate relative changes due to LBNP mediated baroreflex stimulation.

### Statistical analysis

The statistical analysis was carried out using SPSS Statistics (IBM, Version 29.0). Kolmogorov–Smirnov and Levene’s tests were used to assess normality of the distribution and homogeneity for each variable. After a normality of data distribution was confirmed, the baseline clinical and physiological data for FM and HC were analyzed by single-factor variance analysis (ANOVA). Changes over time during baroreflex stimulation were analyzed by repeated measures ANOVA with the between-subject factor ‘disease’ (patients vs. controls). Greenhouse–Geisser correction was used to correct for violation of sphericity. To identify differences during each minute of baroreflex stimulation, t-tests were calculated as post hoc tests. To investigate baseline independent changes induced by baroreflex stimulation, BF was normalized to baseline with BF at baseline being 1. All values are given as means ± standard error (SEM) or medians and IQR in the case of non-normal distribution. Differences were considered significant if *p* < 0.05.

### Ethical approval

All participants provided informed and written consent. The study was conducted in accordance with the declaration of Helsinki. The study was approved by the local ethical committee of the Medical Faculty of the Justus-Liebig-University, Giessen.

## Results

### Cardiovascular parameters

HR, systolic and diastolic BP did not differ between the FM patients and HC neither at baseline nor during LBNP stimulation (For details see Table 1). BRS at baseline was significantly lower in FM compared to healthy controls (F = 14.791; dF = 1,20; *p* < 0.001).

### MSNA

#### Burst frequency (BF)

**Fig. 1:**
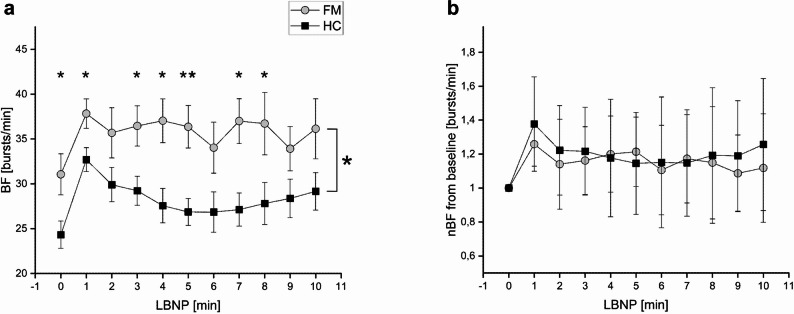
Microneurography results by MSNA burst frequency [BF], FM patients vs. healthy controls. Displays the time course of MSNA in burst frequency (BF; **a**) and normalized burst frequency (nBF; **b**) in FM patients (light grey circles) and healthy controls (filled squares). **a** BF was higher in FM patients compared to the control group at rest (F=5.736; *p*=0.028; ANOVA) and during baroreflex stimulation (F=5.057;*p*=0.044; rm-ANOVA). **b** in addition displays changes in MSNA compared to baseline (resting period) after offset elimination at each time point during LBNP stimulation by BF in the FM patients compared to healthy controls. No significant differences were observed, after normalization of data. Therefore the sympathetic response capability resembles identical between the two groups. For details see Table 2. *: *p*<0.05; **:*p*<0.01. BF = burst frequency; nBF = normalized burst frequency; LBNP: lower-body negative pressure; FM = Fibromyalgia patients; HC = Healthy controls

BF was higher in FM compared to the control group (F = 5.736; dF = 1,20; *p* = 0.028; ANOVA). The MSNA outflow during LBNP stimulation was higher in FM patients compared to the healthy controls (F = 5.057; *p* = 0.044; rm-ANOVA). BF was higher in FM patients not only at baseline, but also during minute 1, 3, 4, 5, 7 and 8 during LBNP stimulation (for details see Table 2 and Fig. 1a)

**Table 2:** MSNA of FM patients and healthy controls. Displays the MSNA data of our FM patients (*n*=10), healthy controls (*n*=11)

		baroreflex stimulation (LBNP)									
	baseline	min 1	min 2	min 3	min 4	min 5	min 6	min 7	min 8	min 9	min 10
Burst frequency (bursts/min)											
FM	31.1 ± 2.3	37.8 ± 1.6	35.7 ± 2.8	36.5 ± 2.2	37.0 ± 2.4	36.4 ± 2.4	34.0 ± 2.9	37.0 ± 2.5	36.7 ± 3.5	33.9 ± 2.5	36.1 ± 3.4
HC	24.3 ± 1.5	32.7 ± 1.3	29.9 ± 1.9	29.2 ± 1.6	27.6 ± 1.9	26.9 ± 1.5	26.9 ± 2.2	27.1 ± 1.9	27.8 ± 2.3	28.4 ± 2.1	29.2 ± 2.1
*p*	0.028*	0.043*	0.136	0.029*	0.014*	0.006**	0.104	0.013*	0.021*	0.090	0.183
Normalized burst frequency (bursts/min)											
FM	1.0 ± 0.0	1.26 ± 0.13	1.14 ± 0.26	1.16 ± 0.20	1.20 ± 0.22	1.21 ± 0.20	1.11 ± 0.26	1.17 ± 0.26	1.15 ± 0.33	1.09 ± 0.23	1.12 ± 0.32
HC	1.0 ± 0.0	1.38 ± 0.28	1.22 ± 0.26	1.22 ± 0.26	1.18 ± 0.35	1.45 ± 0.30	1.15 ± 0.39	1.15 ± 0.31	1.19 ± 0.40	1.19 ± 0.33	1.26 ± 0.39
*p*	--	0.116	0.244	0.298	0.430	0.277	0.384	0.426	0.405	0.242	0.225

*FM* fibromyalgia, *HC* healthy controls

*p<0.05, **p<0.01, the comparison between the patients and the HC is shown

After BF normalization to baseline, the responsiveness of the two groups did not differ during baroreflex stimulation via LBNP (F = 0.080; *p* = 0.781; rm-ANOVA). Additional post-hoc t-test calculations showed no significant difference for the whole stimulation period (Table 2 and Fig. 1b).

#### MSNA and pain rating

The mean NRS pain rating of FM patients at the time of the experiment was 4.89 ± 0.66. Pain intensity significantly correlated with resting MSNA outflow (*r* = 0.760; *p* = 0.018; 95%-CI: [0.23, 0.94]; supplemental Fig. 1).

#### Baroreflex sensitivity, MSNA and pain rating

There was a negative correlation between BRS and BF at baseline (BRS: 5.0 ± 0.5; *r*=−0.792; *p* = 0.019; 95%-CI: [−0.27, −0.92]). BRS showed a tendency to a negative correlation with pain intensity on the NRS (*r*=−0.641; *p* = 0.063; 95%-CI: [−0.01, −0.80]; supplemental Fig. 1).

## Discussion

Fibromyalgia patients suffered from chronic pain and showed increased sympathetic outflow at rest. Pain intensity was positively correlated with resting sympathetic outflow and negatively correlated with BRS. LBNP stimulation further increased sympathetic activity in both investigated groups. While the absolute sympathetic outflow was increased in FM, their relative sympathetic reaction capability to LBNP stimulation was identical to that of HC. Our findings provide evidence for an interconnection between absolute increased sympathetic outflow and pain in FM. Therefore changes in autonomic function play an important role in pain chronification in FM.

The pathophysiology of fibromyalgia involves altered pain processing, an abnormal neuroendocrine component, environmental factors, sleep disturbances, and genetic components as well as autonomic nervous system dysfunction [27]. There is strong evidence of autonomic dysfunction in FM [4], but it is unclear if this is a cause or an effect or both. A review by Martinez-Martinez and colleagues found contradictory results from 70 studies in FM: 47 demonstrated sympathetic predominance, four showed parasympathetic predominance, 10 described autonomic dysfunction in general and nine found no difference between patients and controls [4]. Kadetoff and Kosek stated, that significant heterogeneous autonomic dysfunctions is evident in FM [27, 28].

MSNA measurement provides a direct neuronal signal that serves as a surrogate for sympathetic activity. This is advantageous compared to other autonomic examination parameters which underlie many regulatory mechanisms like BP and HR. There is inconsistent and sparse evidence from baroreflex function in FM as measured by MSNA. The first study to use sympathetic microneurography found no difference in MSNA outflow between FM and HC at baseline or after sympathetic activation [17]. Other investigations have reported increased MSNA outflow in FM at rest, which supports our current results [18, 29]. Previous studies examined the autonomic responses of FM patients during tilt testing and reported no additional increase in MSNA. In contrast, our findings demonstrate a further increase in MSNA during selective baroreflex stimulation by LBNP in FM patients. This suggests that altered baroreflex control and functional changes within the baroreflex pathway contribute to autonomic dysregulation in FM. Several studies have shown a reduction in pain intensity following LBNP stimulation [30]. Comparing our results with previous studies, the LBNP stimulation cannot be directly compared to tilt test, which was applied in most other studies. While tilt test and LBNP both impose orthostatic stress by unloading cardiopulmonary and arterial baroreceptors, the mechanical and sensory components of these interventions differ. LBNP isolated activates baroreceptors, while a tilt test activates baroreceptors, but additionally vestibular and central integrative pathways - engaging both autonomic and somatosensory systems. Tilt test is thereby causing a more abrupt shift in central blood volume and thereby representing a stronger cardiovascular challenge compared to LBNP at equivalent levels of baroreceptor unloading. A stronger intervention is required to stabilize cardiovascular function after this challenge during the tilt test [31]. In our study we analyzed direct sympathetic outflow by MSNA in FM patients, when LBNP induced sympathetic reactivity without confounding systems (vestibular, somatosensory). In FM patients, the expected increase in muscle sympathetic nerve activity (MSNA) during tilt testing may be blunted despite the stronger orthostatic stimulus. This could indicate altered central integration of baroreceptor and vestibular afferents, impaired cardiopulmonary baroreflex sensitivity, or a ceiling effect due to elevated baseline sympathetic tone commonly reported in FM. Such mechanisms may limit further sympathetic activation during orthostatic stress, contributing to the dys-autonomic features and orthostatic intolerance frequently observed in this population.

Baroreflex control has been recently linked to pain in FM. In a controlled intervention study on 32 FM patients, cardiac gated neuromodulation which involved the application of noxious and non-noxious cardiac synchronized electrical stimuli, was capable of increasing the BRS by 41% and decreasing pain by 36%. One possible explanation for this effect is a possible reset of BRS with temporarily restoration of pain inhibition. The observed improvement in BRS during pain inhibition are possibly due to activation of periaqueductal gray or nucleus tractus solitarius, by enhancing cardio-vagal outflow and suppression of sympathetic activity. Additionally, endogenous opioid pathways and attenuation of hypothalamic–pituitary–adrenal axis activity normalize autonomic function and mitigate pain/stress-related blunting of baroreflex control [32]. In a randomized prospective study in 62 FM patients a systolic extinction training increased BRS by 57% and a significant pain reduction was achieved in 82% of FM patients over a 12 month follow period. Therefore, BRS conditioning and resetting might be key factors in restoration of pain inhibition [14].

The association of BRS dysfunction and chronic pain most likely arises from the interaction of both, central and peripheral mechanisms: On the one hand chronic nociceptive input with consecutive central sensitization heighten the excitability of brainstem autonomic nuclei (e.g., the rostral ventrolateral medulla), and impaired baroreflex buffering allows central sympathetic drive to become unrestrained. On the other hand, peripheral sensitization and sympathetic sensory coupling -such as the increased expression of α-adrenergic receptors on nociceptors- foster a self-sustaining loop of pain and sympathetic arousal. Finally, chronic activation of hypothalamic-pituitary-adrenergic axis and neuro-immune inflammation further shift autonomic balance toward sympathetic dominance [33, 34].

An additional aspect of autonomic function and pain perception in FM patients can be derived, when differentiating trait and state reconciliation: Although FM is consistently characterized by increased sympathetic nerve activity at rest (MSNA) and reduced baroreflex sensitivity -an indication of dispositional sympathetic hyperactivity [18, 35]- experimental interventions such as lower body negative pressure (LBNP) stimulation can paradoxically produce analgesic effects. This contradiction can be resolved by differentiating between tonic (dispositional) and phasic (state-dependent) baroreflex modulation: Increased basal MSNA reflects persistent, increased sympathetic tone and reduced tonic inhibition of pain transmission. In contrast, acute baroreceptor activation or unloading during LBNP can activate phasic central gating mechanisms that modulate nociceptive transmission at the brainstem and cortical levels [36]. Thus, even in the presence of chronic sympathetic over activity, short-term baroreflex-induced changes in afferent signaling can temporarily activate descending inhibitory circuits, resulting in short-term analgesia. These findings underscore that autonomic dysregulation in FM involves both a trait component (sustained sympathetic predominance) and a state-dependent dimension, whereby acute baroreflex activation can modulate pain perception through dynamic central integration.

Extending the data from MSNA outflow and BRS restoration, our findings support that decreased BRS and increased pain intensity correlated with MSNA outflow. This corroborates previous findings showing that pain intensity correlated with high sympathetic nerve activity and decreased BRS [19]. Zamuner and colleagues demonstrated that, among other parameters, pain intensity correlated positively with MSNA and inversely with BRS. They concluded that higher sympathetic drive was associated with higher chronic pain.

Our results show that the sympathetic reflex arcs, activated during LBNP stimulation, are intact in FM. Since FM patients are able to not only further increase their sympathetic outflow during baroreflex stimulation but that these patients also presented the identical relative sympathetic response capability as HC. However, sympathetic tone is obviously only part of the puzzle in understanding the pain in FM. Changes in the baroreflex circuit therefore play a role in the pathophysiology of FM. Although sympathetic activity and autonomic dysfunction contribute in the development and maintenance of chronic pain, the restoration of bodily homeostasis and pain inhibition are multidimensional.

### Limitations

The presented study renders some limitations. The number of patients recruited is small. However, we were able to demonstrate increased resting sympathetic outflow in every one of the patients suggesting reliable results. MSNA is a very stable and robust parameter and therefore provides robust data in small cohorts [25, 37].

The washout phase of 72 h for cardiovascular active agents such as beta-blockers and statins was rather short. To adequately exclude pharmacological effects on baroreflex activity, a washout of at least 2 weeks for beta-blockers and 3 weeks for statins would be sufficient. This duration allows both hemodynamic and autonomic parameters to return to physiological baseline before testing but renders a significant increase in risk for cardiovascular events. We therefore decided on a 72 h washout, to establish reduction of immediate medication effects and in the same moment not to increase cardiovascular risk.

## Conclusion

To the best of our knowledge, this is the first study showing decreased BRS combined with increased sympathetic activity of vasoconstrictor outflow as part of baroreflex control with connections to the painfulness of FM. Our data pinpoints to the importance of sympathetic changes in FM. Therefore, further studies are needed to elucidate the role of baroreflex dysfunction in FM that might imply specific therapeutic strategies in the future.

## Supplementary Information

Supplementary Material 1. Displays the correlation of MSNA in burst frequency with the pain rating on the NRS (MSNA BF x Pain NRS; 1.1) and the correlation of baroreflex sensitivity with the pain rating on the NRS (BRS x Pain NRS; Figure 1.2) in 10 FM patients.

Supplementary Material 2. Resting sympathetic outflow as measured by MSNA burst frequency (BF) correlated significantly with the pain intensity rating of 10 FM patients. (*r*=0.760; *p*=0.018; 95%-CI: [0.23, 0.94]); *:*p*<0.05. Supplemental Figure 1.2: BRS showed a tendency towards a negative correlation with pain intensity rating on the NRS (*r*=-0.641; *p*=0.063; 95%-CI: [-0.01, -0.80]).

## Data Availability

All data supporting this study are available through the corresponding author on request.

## Abbreviations

ANOVA: analysis of variance
BF: burst frequency
BRS: baroreflex sensitivity
DBP: diastolic blood pressure
dACC: dorsal anterior cingulate cortex
dlPFC: dorsolateral prefrontal cortex
NTS: dorsal medial nucleus tractus solitaries
FM: fibromyalgia
HC: healthy controls
HR: heart rate
LBNP: lower body negative pressure
MSNA: muscle sympathetic nerve activity
NRS: numeric rating scale
PAG: periaqueductal gray
SBP: systolic blood pressure
SEM: standard error of mean
