# Supplementary figures and images for: Association between altered baroreflex control and pain intensity in fibromyalgia
Source: BMC Neurol. 2025 Dec 5;25:498. doi: 10.1186/s12883-025-04566-x (PMC12699904; doi:10.1186/s12883-025-04566-x)

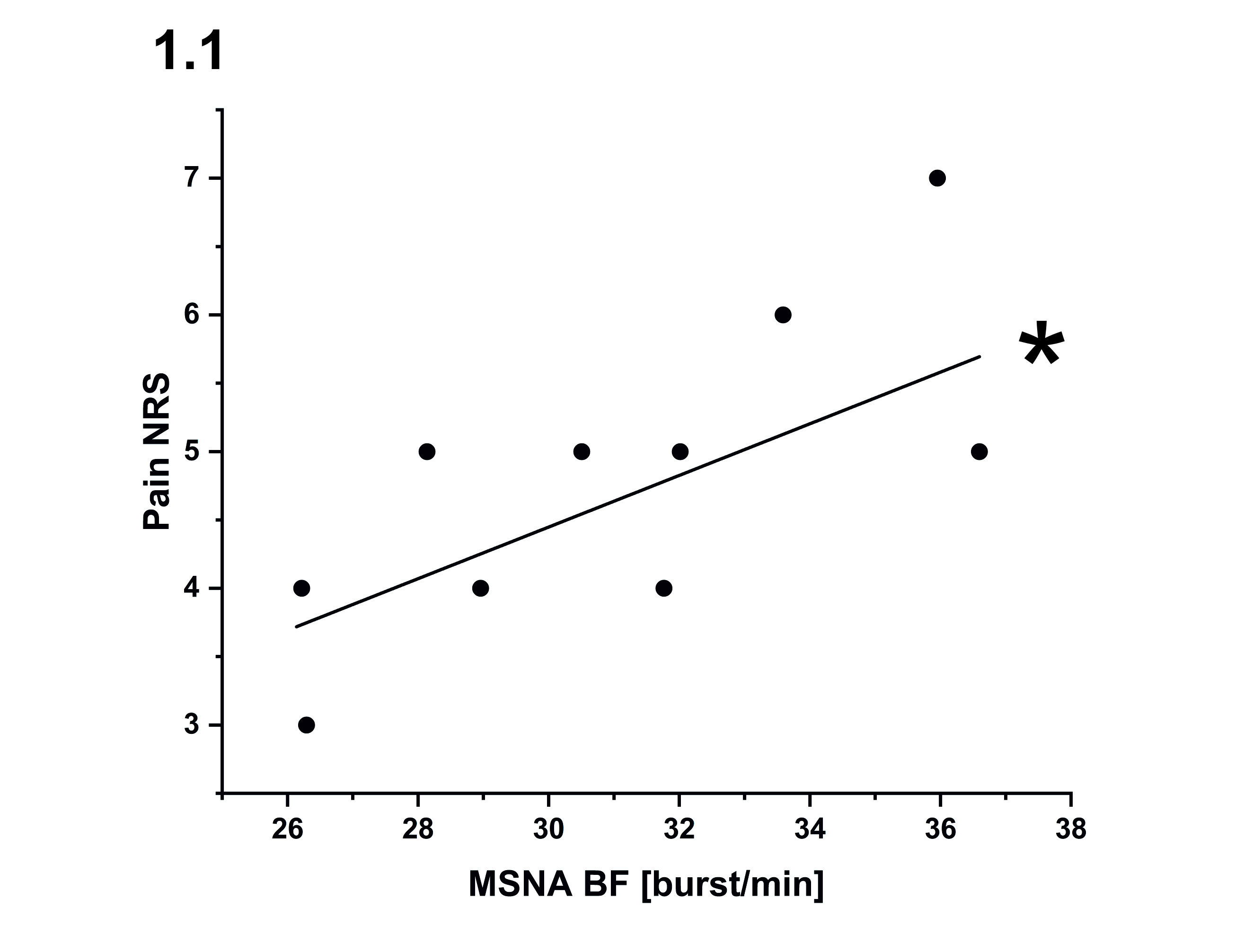

Supplement: Supplementary file 1 — Supplementary Material 1. Displays the correlation of MSNA in burst frequency with the pain rating on the NRS (MSNA BF x Pain NRS; 1.1) and the correlation of baroreflex sensitivity with the pain rating on the NRS (BRS x Pain NRS; Figure 1.2) in 10 FM patients. [file 12883_2025_4566_MOESM1_ESM.jpg]

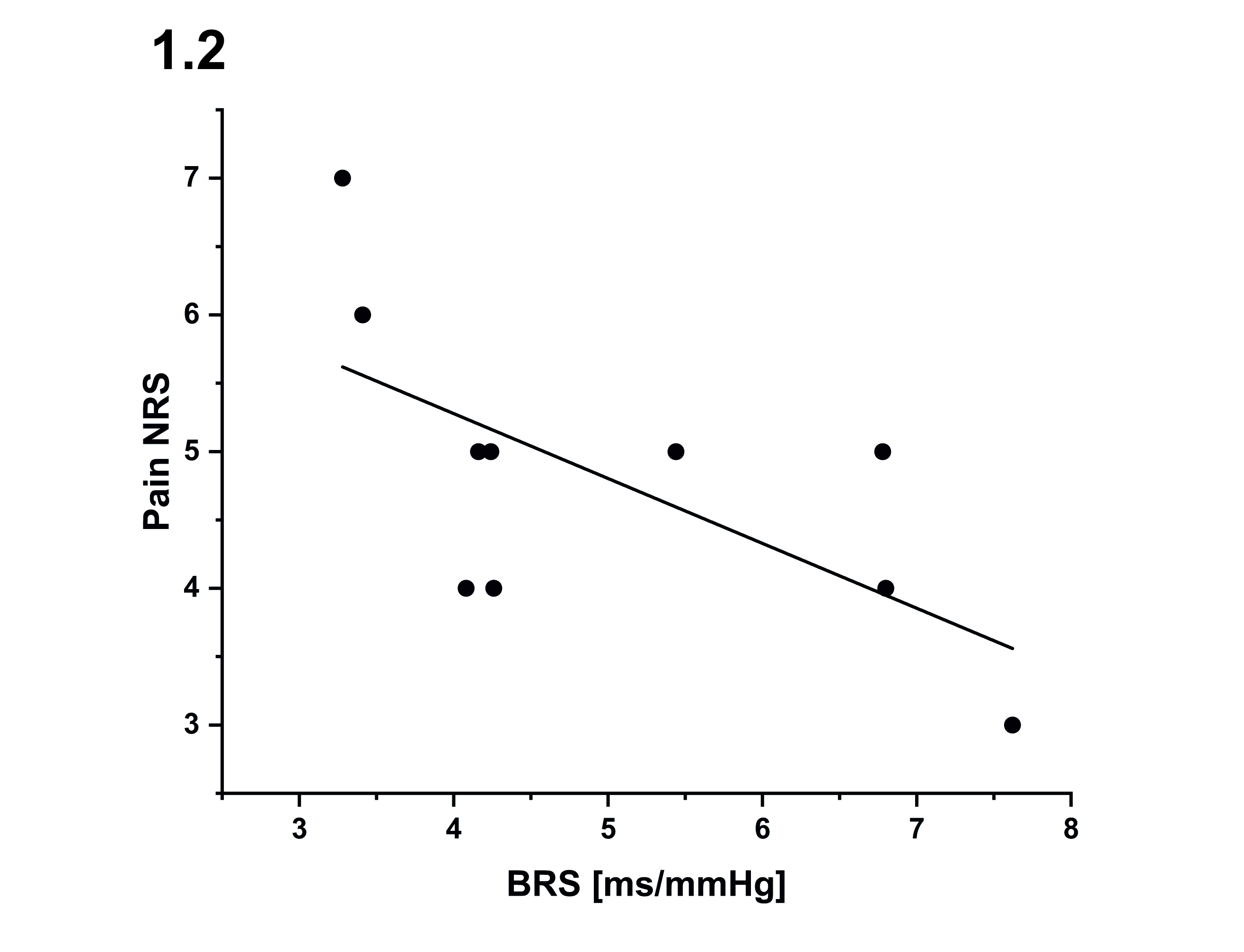

Supplement: Supplementary file 2 — Supplementary Material 2. Resting sympathetic outflow as measured by MSNA burst frequency (BF) correlated significantly with the pain intensity rating of 10 FM patients. (r=0.760; p=0.018; 95%-CI: [0.23, 0.94]); *:p<0.05. Supplemental Figure 1.2: BRS showed a tendency towards a negative correlation with pain intensity rating on the NRS (r=-0.641; p=0.063; 95%-CI: [-0.01, -0.80]). [file 12883_2025_4566_MOESM2_ESM.jpg]
